# Identification of pear tissue mechanical parameters by integrating machine learning and optimization methods

**DOI:** 10.1371/journal.pone.0353997

**Published:** 2026-08-14

**Authors:** Yuanyuan Dai, Fei Meng, Xiaohua Xie, Yangyang Liu

**Affiliations:** 1 School of Food and Environmental Engineering, Chuzhou Vocational and Technical College, Chuzhou, China; 2 School of Mechanical Engineering, Anhui University of Technology, Ma’anshan, China; 3 School of Engineering, Anhui Agricultural University, Hefei, China; King Mongkut’s University of Technology North Bangkok, THAILAND

## Abstract

The mechanical properties of fruit tissue play a crucial role in various food processing activities, including sorting, packaging, and transportation. Most traditional testing processes are destructive, time-consuming, and cannot capture the mechanical response under loading conditions. The present research proposes a parameter identification model that combines experimental analysis, finite element analysis, and a hybrid machine learning model to describe the viscoelastic behaviour of pear tissue. To obtain a rapid mapping from the intrinsic material constants to mechanical behavior, we develop a feasible surrogate architecture. This framework incorporates convolutional layers to extract spatial features, along with an attention-based bidirectional LSTM, which ensures a high-fidelity approximation of mechanical behavior. An enhanced genetic algorithm is suggested as a solution to the identification problem. The framework is tested through compression tests on Huangguan pears. The results indicate that the mean absolute percentage error of the surrogate model can reach a minimum of 0.05, and the force-displacement plots show an error of less than 0.08 compared to experimental data. The suggested technique minimizes the need for extensive physical testing while preserving predictive accuracy, providing a computationally efficient and experimentally economical solution for determining the material parameters of fruit tissues. It has the potential to assist in designing and optimizing food protection equipment for transporting fresh fruits, thereby reducing storage losses.

## 1. Introduction

Mechanical sorting, packaging, and transportation cause external forces on fruits that may result in tissue damage and quality loss [1,2]. Even a small injury can cause visible bruising and quick decay in pears and influence customer acceptance and economic value. The mechanical behavior of fruit tissues in these processing-related loading conditions is thus important in designing optimum equipment and minimizing postharvest losses. Traditional ways of estimating mechanical properties usually involve destructive tests and empirical constitutive equations. They require a large number of samples, and cannot be easily applied to online processing situations [3–9]. Recent advances in deep learning provide an opportunity to overcome such challenges. Neural networks have the ability to learn highly nonlinear interactions among high-dimensional experimental data without a predefined mathematical structure and hold great potential in material modeling and parameter identification [10]. Deep learning application to the mechanical research of pears might assist in decreasing postharvest damage, optimizing the supply chain, increasing product quality, and ensuring industry sustainability [11–13].

The cell structure, tissue composition, and physiological condition govern the mechanical behavior of pears. The flesh exhibits typical viscoelastic solid properties when it is loaded under quasi-static conditions. Stress relaxation tests suggest that the rheological behavior of the flesh can be modeled by combinations of elastic and viscous components, including the Maxwell or generalized Maxwell models [14]. The mechanical properties of pears change dynamically during ripening and postharvest maturation. Generally, as maturity increases, degradation of cell wall components and weakening of intercellular adhesion lead to decreased fruit firmness and elastic modulus, making the fruit more susceptible to mechanical damage [15]. Classical parameter identification methods are based on continuum mechanics theory. However, constructing precise analytical models for highly nonlinear damage behaviors is itself a challenge. To meet the demand for online, non-destructive testing, acoustic, optical, and electromagnetic techniques have been extensively explored [16,17]. Nevertheless, these approaches are often based on empirical black-box correlations and lack a clear physical basis. Moreover, models such as PLS regression and SVM struggle to generalize when handling nonlinear problems with significant individual variation and complex causal influences [18].

In general, a feedforward neural network comprising one or more hidden layers can model any continuous function arbitrarily precisely, thus serving as an attractive tool to build input‑output mappings for complex nonlinear systems [19]. In computational mechanics, neural networks can be directly used as material constitutive models [20]. Compared to traditional models, neural network constitutive models do not require predefined specific functional forms, only sufficient training data covering the material’s behavior space. Another common paradigm uses neural networks as surrogate models to accelerate computationally expensive parameter inversion processes. Specifically, a finite element model is first used to generate a large amount of simulation data under different parameter combinations; subsequently, a neural network is trained to learn the inverse mapping from mechanical responses to material parameters [21]. Once this network has been trained, actual measured response data can be placed into it to invert the corresponding mechanical parameters in real-time in a fast manner. Such an approach substitutes the computationally intensive forward problem solving with an offline phase, which is run once only, with a single effective forward propagation performed in the online stage. The mechanical response of fruit is basically a loading history function. RNNs and various related models are specifically trained to work with sequence data and long-term interactions, which makes them quite appropriate to model rate-dependent behavior and stress relaxation properties of viscoelastic materials [22]. For problems involving spatial field information, convolutional neural networks are favored for their powerful spatial feature extraction capabilities [23]. One-dimensional convolutional neural networks have been successfully applied to process vibration spectrum data of fruits for predicting indicators like firmness [24]. Purely data-driven neural networks might contradict well-known laws of physics and often need large volumes of training data. The physics-informed neural networks tackle this issue by incorporating governing equations into the loss function as regularization, constraining network predictions to obey physical rules [25].The paradigm of physics-guided machine learning is much more efficient in terms of data and more accurate in terms of model generalization and extrapolation reliability, which represents a good opportunity in developing high-fidelity mechanical parameter identification models using small sets of high-quality experimental data, even though it has not yet been used in agricultural biomaterials applications [26]. From an engineering perspective, these physics-informed methodologies provide a reliable way to identify parameters that can be incorporated into the design process of fruit protection devices.

To this end, the present study aims to establish an efficient mechanical parameter identification strategy that integrates experimental testing, finite element simulation, and machine learning to overcome the limitations of conventional destructive methods. Unlike existing surrogate‑based parameter identification methods that rely on standard RNNs or simple feedforward networks, the proposed CNN-BiLSTM-Attention model uniquely captures both local spatial features and bidirectional temporal dependencies in viscoelastic responses. Furthermore, the improved genetic algorithm (IGA) introduces a three‑layer hybrid initialization, dynamic fitness‑based subgroup evolution, and an adaptive diversity adjustment mechanism, which significantly enhances convergence speed and global search capability on high‑dimensional material parameter spaces.

## 2. Materials and methods

### 2.1. Parameter identification

**Fig 1:**
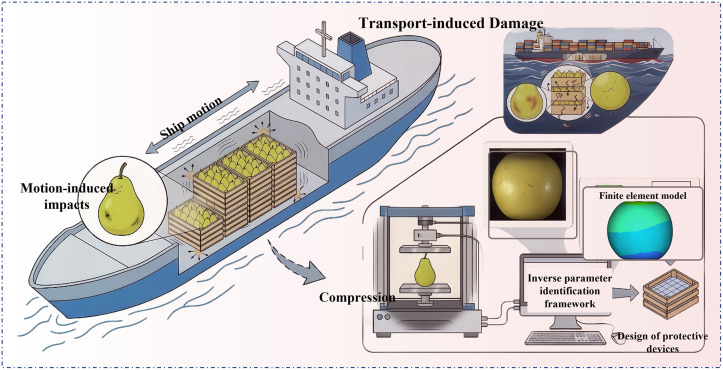
Description of low-speed damage scenarios of pears during transportation.

Similar to other fruits, in the course of transportation and storage, pears may be damaged mechanically, including the effects of falling and mechanical extrusion [27–30]. Small injuries may cause a reduction in the quality of fruits and the development of decay. Taking transportation as an example, Fig 1 shows a schematic diagram of pear damage protection during long-distance cargo ship transportation. In order to minimize pear damage as much as possible, a protective device with buffering effect needs to be designed. However, to efficiently protect fruits, it is necessary to establish a high-precision fruit simulation model. Hence, there is a need to carry out a study regarding mechanical damage to fruits. To determine the mechanical parameters of pears, the given study establishes a data-driven method to calibrate the minor damage model of pears under potential mechanical loads.

The proposed approach starts by modelling the pear by creating its finite element representation. Only a small number of low‑cost experiments are performed to obtain the mechanical response data, i.e., compression load‑displacement curves. The findings of these experiments are the starting point of developing a machine learning based surrogate model. In particular, it is through this data that a predictive model is trained to make predictions of the mechanical response of the pear in a more efficient and accurate manner when presented with certain material parameters. Having such a surrogate model, the approach uses an algorithm that searches the parameter space. The optimisation process is expected to reduce the difference between the numerical surrogate model and the experimental result. With repeated changes in the material parameters, the error is reduced to a reasonable level. This is to make sure that the parameters identified are a good representation of the mechanical properties. The proposed method is not only very accurate, but also minimizes the use of elaborate, expensive, and time-consuming testing that involves experimentation. Although validated on pears, the framework is not limited to this specific fruit or loading condition. The combination of finite element simulation, surrogate modeling, and global optimization can be adapted to other biomaterials and processing scenarios where mechanical interactions between product and equipment influence final quality.

### 2.2. Predictive model

Sequence data prediction is an important research direction in data science and machine learning, widely applied in fields such as finance, meteorology, energy, and transportation. Traditional approaches like autoregressive moving average and its integrated variant are limited when applied to nonlinear, non-stationary time series [31–33]. With the development of deep learning techniques, neural network-based prediction methods demonstrate powerful nonlinear modeling capabilities. Convolutional neural networks (CNNs) specialize in spatial feature extraction, whereas long short-term memory networks (LSTMs) are adept at modeling long-range sequential dependencies. However, a single model often struggles to simultaneously handle spatiotemporal features and long-range dependencies. Therefore, this work develops a CNN-BiLSTM-Attention hybrid prediction model [34,35]. Here, the framework of the network is designed based on the training sample point data in this study, and some major network parameters can be adjusted as necessary according to the complexity of the problem and the sample data.

The framework for sequence data prediction combines CNN-based local feature extraction, BiLSTM-based bidirectional temporal modeling, and attention-based feature selection. The proposed CNN-BiLSTM-Attention model is composed of a sequence of processing stages: beginning with data preprocessing, followed by feature extraction (CNN), attention-based feature weighting, bidirectional temporal modeling (BiLSTM), and culminating in a prediction output layer. The prediction model takes four material parameters as input. After normalization, they first go into a convolutional feature extraction module. Since the input dimensionality is low, the convolution part uses a lightweight two-layer structure, with kernel size set to 2 × 1 and the number of kernels being 16 and 32 respectively. A ReLU activation function is added after each convolutional layer to enhance nonlinear expressiveness. Then, an SE type channel attention mechanism adaptively weights the convolutional features. After global average pooling, two fully connected layers with 8 and 32 neurons are used to highlight key channel information and suppress redundant features. The attention-weighted features are then fed into a BiLSTM network with 16 hidden units, which captures bidirectional dependencies among deep features and improves the modeling of the overall trend of the load curve. Finally, a fully connected layer with 10 neurons outputs the load values at 10 discrete time points, achieving complete prediction of the compression load curve. To improve convergence stability and prediction accuracy, the CNN-BiLSTM-Attention model is trained with a piecewise learning rate decay strategy and the Adam algorithm.

The computational flow of the model can be formally expressed as:


$$\begin{array}{c} \hat{Y}=W_{fc}\cdot BiLSTM\left(SE\left(CNN(X)\right)\right)+b_{fc} \end{array}$$ (1)

where X represents the input data, and $\hat{Y}$ represents the predicted output. $W_{fc}$ represents the weight of the fully connected layer, and $b_{fc}$ represents the bias of the fully connected layer. The input data is first normalized, mapping the original data to the [0,1] interval to eliminate scale effects and accelerate model convergence:


$$\begin{array}{c} x^{\prime }=\frac{x-x_{\text{min}}}{x_{\text{max}}-x_{\text{min}}} \end{array}$$ (2)

where x represents the original data. After normalization, the data is reshaped into a multidimensional tensor form:


$$\begin{array}{c} X\in R^{N\times C\times H\times W} \end{array}$$ (3)

where N is the number of samples, C denotes the number of channels, and H and W represent the spatial height and width dimensions, respectively. A sequence folding layer then converts this spatial representation into a sequence of feature vectors, enabling the subsequent temporal modeling by the BiLSTM network. The loss function is:


$$\begin{array}{c} Loss=\frac{\text{1}}{N}\sum_{i=\text{1}}^{N}{\left(y_{i}-{\hat{y}}_{i}\right)}^{\text{2}} \end{array}$$ (4)

In Equation 4, $y_{i}$ represents the true value, ${\hat{y}}_{i}$ represents the estimated value. To assess the performance in a comprehensive way, the following evaluation measures are used in this work:


$$\begin{array}{c} \left\{ \begin{array}{c} MAPE=\frac{\text{1}}{N}\sum_{i=\text{1}}^{N}\left|\frac{y_{i}-{\hat{y}}_{i}}{y_{i}}\right|\\ R^{\text{2}}=\text{1}-\frac{\sum_{i=\text{1}}^{N}{\left(y_{i}-{\hat{y}}_{i}\right)}^{\text{2}}}{\sum_{i=\text{1}}^{N}{\left(y_{i}-\overset{―}{y}\right)}^{\text{2}}} \end{array}\right. \end{array}$$ (5)

where *MAPE* is the mean absolute percentage error and *R*² is the coefficient of determination [36,37]. The CNN module is based on a two-layer convolutional architecture for local feature extraction. The first convolutional layer is characterized by:


$$\begin{array}{c} Z_{\text{1}}=ReLU\left(W_{\text{1}}*X+b_{\text{1}}\right) \end{array}$$ (6)

where * denotes the convolution operation. More detailed features are further elicited:


$$\begin{array}{c} Z_{\text{2}}=ReLU\left(W_{\text{2}}*Z_{\text{1}}+b_{\text{2}}\right) \end{array}$$ (7)

SE (Squeeze-and-Excitation) attention mechanism: This mechanism is an adaptive mechanism of recalibration of feature responses by interdependency modeling across channels [38]. In this module, there are three important steps, which include the Squeeze operation, the Excitation operation, and the Reweight operation. The Squeeze operation derives statistics channel wise through global average pooling:


$$\begin{array}{c} z_{c}=\frac{\text{1}}{H\times W}\sum_{i=\text{1}}^{H}\sum_{j=\text{1}}^{W}u_{c}(i,j) \end{array}$$ (8)

where $u_{c}$ represents the feature of the c-th channel.

This study uses MATLAB to establish a CNN-BiLSTM-Attention prediction model and reads the training data [39]. The experimental data used for training and validation includes force–displacement curves, capturing the viscoelastic behavior of pear tissue. The input features to the CNN-BiLSTM-Attention model consist of four viscoelastic model parameters: instantaneous shear modulus (*G*_0_), relaxation rate constant (*β*), long-term shear modulus (*G*_∞_), and bulk modulus (*K*). The output is the predicted force-displacement curve under a specified compression condition. The viscoelastic behavior is described by a generalized Maxwell model, where the shear relaxation modulus is expressed as [40]:


$$\begin{array}{c} G(t)=G_{\infty }+(G_{\text{0}}-G_{\infty })\text{exp}(-\beta t) \end{array}$$ (9)

Here, the relaxation time constant *β*= $\text{1}/\tau_{\text{1}}$. The pear flesh is assumed to be a homogeneous, isotropic, and linearly viscoelastic material under quasi‑static compression. The shear relaxation behavior is described by a single‑branch generalized Maxwell model.

### 2.3. Optimization algorithm

The paper presents an improved genetic algorithm (IGA), which is aimed at solving single-objective optimization problems in high-dimensional parameter spaces. It is well known that classical genetic algorithms (GA) suffer from various drawbacks such as slow convergence, vulnerability to local optima, and inability to explore and exploit high-dimensional optimization problems [41–43]. To overcome these issues, the proposed IGA introduces several enhancements, including a three-layer hybrid initialization scheme, a dynamic grouping algorithm, and adaptive approach selection to improve performance. IGA divides the population into three classes consisting of elite, medium, and exploration and applies different evolution strategies to each group to strike a dynamic equilibrium between exploration and exploitation. The initial population is generated using the three-layer hybrid strategy in the cyclic process. In the evaluation stage, the fitness value of every individual is estimated. These fitness values are used to dynamically divide the population into three subgroups by iterative optimization where each subgroup uses a different evolutionary strategy. The periodic adaptive modifications are also employed to guarantee the ongoing effectiveness of optimization. The algorithm stops at max iteration or convergence, returning the optimal solution and fitness value. This optimization problem can be regarded as minimization problem. Fitness function is the mean square error between the experimental force-displacement curve and the curve forecasted by the CNN-BiLSTM-Attention surrogate model


$$\begin{array}{c} f\left(𝐱\right)=\frac{\text{1}}{N_{t}}\sum_{k=\text{1}}^{N_{t}}{\left(F_{exp}\left(t_{k}\right)-F_{pred}\left(t_{k};𝐱\right)\right)}^{\text{2}} \end{array}$$ (10)

where 𝐱 denotes the four viscoelastic parameters to be identified, $N_{t}$ is the number of time steps. $F_{exp}$ and $F_{pred}$ are experimental and predictive data, respectively. Classical genetic algorithms normally employ randomization of starting points and therefore the distribution of population is uneven. The improved GA uses a three-layer hybrid initialization strategy:


$$\begin{array}{c} \left\{ \begin{array}{c} {\text{P}}_{\text{1}}:{𝐱}_{i}=\text{L}+\left(𝐔-𝐋\right)⊙{𝐇}_{i}\;\;\left(\text{Latin Hypercube Sampling},\text{4}\text{0}\%\right)\\ {\text{P}}_{\text{2}}:{𝐱}_{i}=\text{L}+\left(𝐔-𝐋\right)⊙{𝐂}_{i}\;\;\left(\text{Chaotic Mapping},\text{4}\text{0}\%\right)\\ {\text{P}}_{\text{3}}:{𝐱}_{i}={𝐱}_{\text{base}}+\sigma ⊙𝐍(\text{0},\text{1})\;\;\left(\text{Gaussian Perturbation},\text{2}\text{0}\%\right) \end{array}\right. \end{array}$$ (11)

where 𝐇 is a Latin Hypercube sequence, 𝐂 is a chaotic sequence, and σ=0.1(𝐔−𝐋) is the perturbation strength.

The improved GA dynamically subdivides the population into three groups according to the fitness of an individual:


$$\begin{array}{c} \left\{ \begin{array}{c} G_{\text{elite}}=\left\{{𝐱}_{i}:rank\left(f\left({𝐱}_{i}\right)\right)\leq \text{0}.\text{2}N\right\}\\ G_{\text{intermediate}}=\left\{{𝐱}_{i}:\text{0}.\text{2}N<rank\left(f\left({𝐱}_{i}\right)\right)\leq \text{0}.\text{8}N\right\}\\ G_{\text{exploration}}=\left\{{𝐱}_{i}:rank\left(f\left({𝐱}_{i}\right)\right)>\text{0}.\text{8}N\right\} \end{array}\right. \end{array}$$ (12)

where N denotes the population size, and rank(·) is the fitness ranking function. The improved GA makes use of group-specific evolutionary strategies: Elite Group (20%): Used for local refinement: ${𝐱}_{\text{new}}={𝐱}_{\text{old}}+\alpha \left({𝐱}_{\text{best}}-{𝐱}_{\text{old}}\right)$; Intermediate Group (60%): Used for adaptive crossover and mutation: $P_{c}=\text{0}.\text{8}-\text{0}.\text{3}t$, $P_{m}=\text{0}.\text{1}+\text{0}.\text{2}(\text{1}-t)$; Exploration Group (20%): Used for global exploration: ${𝐱}_{\text{new}}=𝐋+\left(𝐔-𝐋\right)⊙𝐑$. Here, t is the normalized iteration progress, α is the learning rate, and 𝐑 is a random vector. The improved GA incorporates an adaptive balancing adjustment mechanism. The algorithm periodically monitors population diversity and dynamically adjusts the balance between exploration and exploitation:


$$\begin{array}{c} \left\{ \begin{array}{c} \text{Diversity metric}:\;D=\frac{\sigma (f)}{\mu (f)+\epsilon }\\ \text{Adjustment condition}:\;\text{If}\;D<{\text{1}\text{0}}^{-\text{4}}\;\text{and}\;t>\text{0}.\text{3}\\ \text{Adjustment operation}:\;\text{Increase}\;\text{3}\text{0}\%\;\text{of exploratory individuals} \end{array}\right. \end{array}$$ (13)

The performance of IGA, standard GA, and standard PSO is evaluated on four classical test functions [44]::


$$\begin{array}{c} \left\{ \begin{array}{c} @c\text{Sphere}:f\left(𝐱\right)=\sum_{i=\text{1}}^{D}x_{i}^{\text{2}}\\ \text{Rastrigin}:f\left(𝐱\right)=\text{1}\text{0}D+\sum_{i=\text{1}}^{D}\left[x_{i}^{\text{2}}-\text{1}\text{0}\text{cos}\left(\text{2}\pi x_{i}\right)\right]\\ \begin{array}{c} \text{Ackley}:f\left(𝐱\right)=-\text{2}\text{0}\text{exp}\left(-\text{0}.\text{2}\sqrt{\frac{\text{1}}{D}\sum_{i=\text{1}}^{D}x_{i}^{\text{2}}}\right)-\text{exp}\left(\frac{\text{1}}{D}\sum_{i=\text{1}}^{D}\text{cos}\left(\text{2}\pi x_{i}\right)\right)+\text{2}\text{0}+e\\ \text{Rosenbrock}:f\left(𝐱\right)=\sum_{i=\text{1}}^{D-\text{1}}\left[\text{1}\text{0}\text{0}{\left(x_{i+\text{1}}-x_{i}^{\text{2}}\right)}^{\text{2}}+{\left(\text{1}-x_{i}\right)}^{\text{2}}\right] \end{array} \end{array}\right. \end{array}$$ (14)

**Fig 2:**
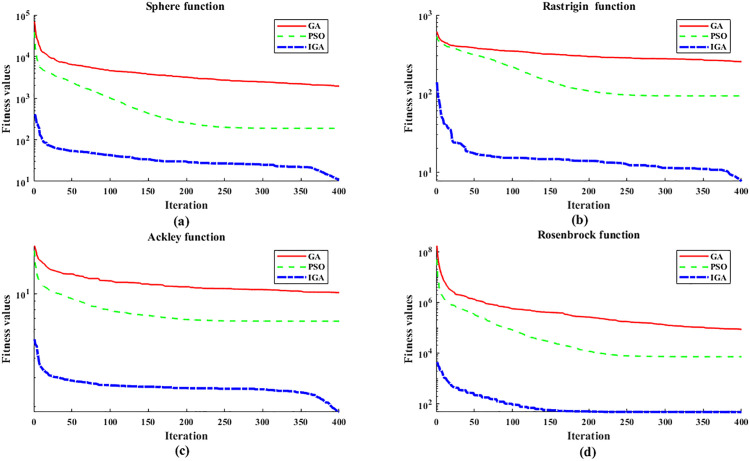
Iterative process of optimization algorithms: (a) Sphere function; (b) Rastrigin function; (c) Ackley function; (d) Rosenbrock function.

**Fig 3:**
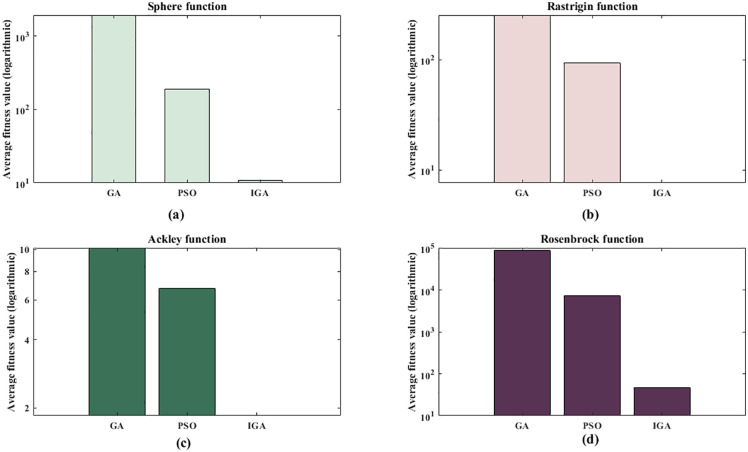
Average fitness function values: (a) Sphere function; (b) Rastrigin function; (c) Ackley function; (d) Rosenbrock function.

For the selected high-dimensional test functions (including Sphere, Rastrigin, Ackley, and Rosenbrock functions), this experiment uniformly sets the problem dimension *D* = 50, the population size *N* = 80, and the maximum iteration number is 400, ensuring that the algorithms have sufficient search and convergence space. As shown in the iterative curves in Fig 2, the IGA exhibits a faster declining trend and a smoother convergence pattern during the evolutionary process. Fig 3 further confirms the superiority of IGA across multiple test functions through the changes in average fitness values. Compared with the general Particle Swarm Optimization (PSO) and GA, IGA not only achieves significantly higher final convergence accuracy but also demonstrates noticeably faster convergence speed, along with stronger stability and robustness over multiple independent runs. In comparison, with traditional GA, IGA minimizes the fitness values of Sphere, Rastrigin, Ackley, and Rosenbrock functions by 99.44%, 97.01%, 81.89%, and 99.95% respectively when minimizing the fitness. Similarly, for the Sphere, Rastrigin, Ackley, and Rosenbrock functions, IGA reduces the fitness values by 94.25%, 91.84%, 72.73%, and 99.34%, respectively, as opposed to the traditional PSO. These findings show that the three-layer hybrid initialization strategy, dynamic grouping mechanism, and real-time adaptive updating strategy used in IGA are good at balancing global exploration and local exploitation resulting in excellent overall performance on high-dimensional complex optimisation problems. This offers it a solid foundation to be used in real world high-dimensional optimization problems such as the identification of mechanical parameters for pears.

## 3. Results and discussion

### 3.1. Experiment and simulation

**Fig 4:**
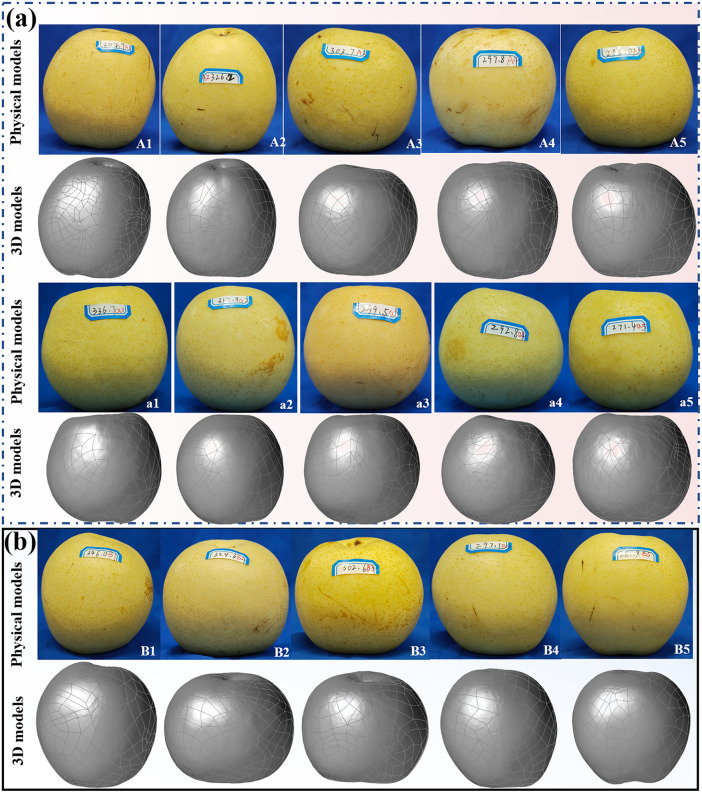
Physical samples: (a) Samples for generating finite element data during parameter identification; (b) Samples for testing the accuracy of parameter identification.

All Huangguan pears used in this study are selected from those harvested at commercial maturity from Dangshan County, Anhui Province, China. Before testing, representative samples are assessed for firmness and soluble solids content (SSC) to confirm uniformity. The pear tissue is modeled as a homogeneous, isotropic viscoelastic material, with key parameters including the *G*_0_, *G*_∞_, *β*, and *K*. Fifteen pears from the same season are selected. All samples are first scanned with a 3D contour scanner to reconstruct their numerical models (as illustrated in Fig 4). Among the experimental samples, one subset is used to establish finite element models for predictive model training, and the other subset is used to validate the feasibility of the identified material parameters. 3D contour scanning technology is commonly used for establishing fruit models to make them more in line with real physical models [45,46]. Pears labeled A1 to A5 and a1 to a5 are used for parameter identification, while pears B1 to B5 are reserved for validating the identified parameters.

**Fig 5:**
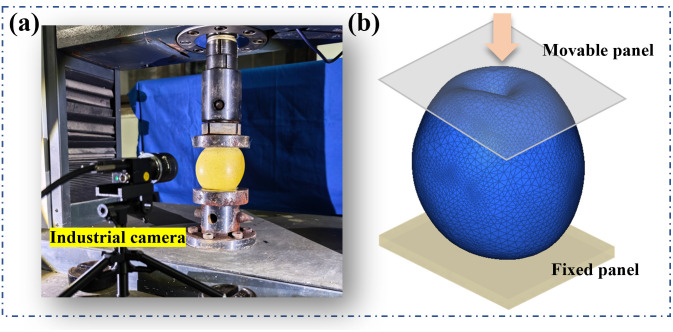
Experimental and simulation model: (a) The experimental instruments; (b) The finite element model.

Uniaxial compression tests are conducted using a material testing machine at a rate of 5 mm/min, with the pear stem placed against the moving upper platen, as shown in Fig 5(a). Correspondingly, the finite element model is established on the LS-DYNA platform (Fig 5(b)), simulating compression between a fixed and a movable rigid plate. The pear geometry is discretized using solid elements and is assigned the *MAT_VISCOELASTIC material model to capture its viscoelastic response [47,48]. The model can be used to explain simultaneously the instantaneous effects of elastic response and viscous flow of the flesh and thus be more representative of the internal deformation of the flesh during harvesting, storage, and transportation. This gives a specific simulation foundation to the damage reduction design and equipment parameters optimization. In this case, the flesh and peel are considered isotropic materials. The size and the type of elements in the finite element model meshing are an important factor in determining the accuracy of the simulations. A suitable mesh size not only guarantees an accurate model but also enhances the level of computational efficiency. In the finite element model (LS‑DYNA), the pear is positioned between a stationary lower rigid plate and a movable upper rigid plate. The upper plate moves downward at a constant velocity of 5 mm/min, and the contact friction is defined. The bottom plate is fully fixed. The pear geometry is discretized with 1 mm solid elements after mesh convergence analysis.

**Fig 6:**
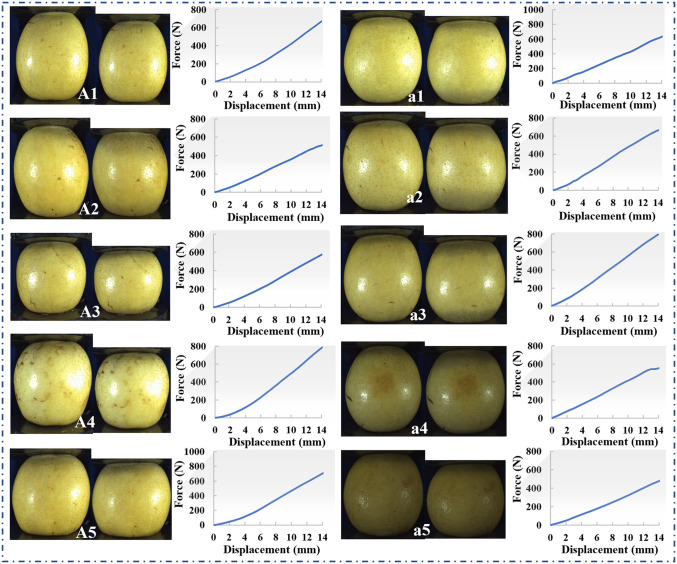
Experiment for generating finite element sample points.

The training data used in this study are all from the same origin and are gathered through random sampling. The compression tests on all pears are shown in Fig 6 by using a universal testing machine. During the early compression stage, the pears show no visible cracking, and the main deformation occurs within the flesh. It can be observed that, during the initial compression stage of pears, the load curves of all samples show a relatively small slope at the beginning, appearing slightly convex upward. The load increases slowly, while the displacement changes significantly. At this stage, this is primarily due to the gentle closure of the porous internal structure of the pear material, such as the intercellular spaces, which allows the pear cell walls to undergo elastic bending easily, resulting in an overall compliant behavior. However, as compression continues, the slope of the load curve, that is, the stiffness, increases steadily, entering a nearly linear stage. At this point, the pear cell walls become sufficiently compacted and begin to bear the main load, exhibiting characteristics similar to the elasticity of a solid. These data will be used separately for the subsequent identification of material parameters.

### 3.2. Parameter calibration results

**Fig 7:**
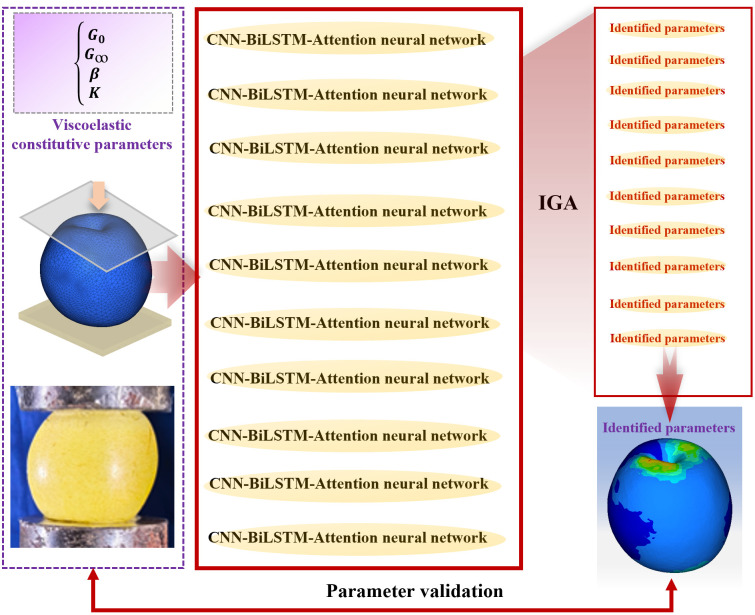
Parameter identification process for Huangguan pears.

**Fig 8:**
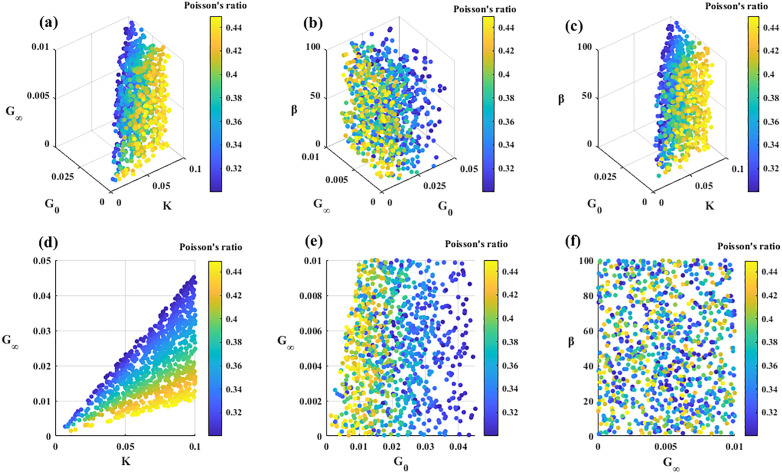
Sample points obtained using optimal Latin hypercube sampling: (a) *G*_0_, *G*_∞_, *K*; (b) (c) *G*_0_, *G*_∞_, *β*; (d) *G*_0_, *K*, *β*; (e) *G*_0_, *G*_∞_; (f) *G*_∞_, *β.*

During the training process, a total of 10000 numerical simulation samples are constructed for model training, based on 10 sets of experimental data. We obtained a final set of viscoelastic constitutive parameters for this variety of Huangguan pears by averaging the material parameters inverted from each experimental dataset. The overall training framework is shown in Fig 7. All pears used in the training phase are from the same season and origin. For each different pear size, 1000 sample points are generated. These points are sampled using the optimal Latin hypercube method, and the sampling space is required to satisfy fundamental viscoelastic mechanical constraints [49]. Based on the constraint relationship between viscoelastic constitutive parameters, assuming the Poisson’s ratio of the Huangguan pear is between 0.3 and 0.45, the obtained sample point data is shown in Fig 8. In this simulation on the LS-DYNA platform, the unit system of material mechanics is defined as GPa. Among these parameters, *K* is greater than *G*_0_, and *G*_0_ is greater than *G*_∞_. The Poisson’s ratio of pear material is mainly influenced by bulk modulus, instantaneous shear modulus, and long-term shear modulus. The measured data from a given experiment are input into the trained predictive model to obtain the mechanical parameters for that specific pear contour type. For a single physical pear prototype, the dataset is randomly split into training (70%), validation (15%), and test (15%). Input features (G₀, G_∞_, β, K) are normalized to [0,1]. Finally, after averaging the 10 sets of parameters, they are substituted into another 5 sets of independent experimental data for validation.

**Fig 9:**
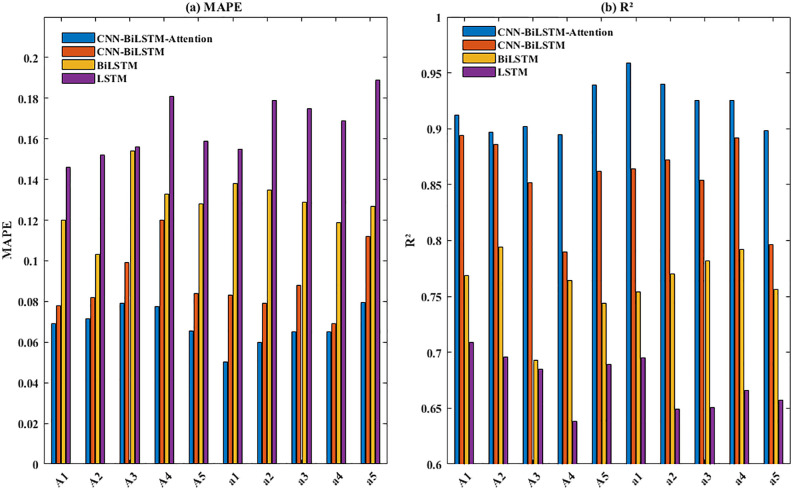
Accuracy evaluation of predictive models: (a) *MAPE*; (b) *R*^2^.

The CNN-BiLSTM-Attention surrogate model takes four viscoelastic model parameters (*G*_0_, *G*_∞_, *β*, *K*) as input and predicts the force-displacement curve. The IGA iteratively searches for the optimal parameters by minimizing the error between predicted and experimental curves. This surrogate model thus learns the mapping from viscoelastic parameters to mechanical response, enabling rapid evaluation during the inverse optimization process. Fig 9 shows the statistical information of *MAPE* for the prediction models of A1 ~ A5 and a1 ~ a5 pears. It can be observed that the *MAPE* is between 0.05 and 0.08, indicating that the prediction error is generally low and stable. The *R*² distribution of all prediction models is in the high range of 0.90 ~ 0.96, further indicating that all prediction models have excellent fitting ability. Compared with conventional LSTM, BiLSTM, and CNN-BiLSTM, the CNN-BiLSTM-Attention model exhibits a lower overall *MAPE* and a higher overall *R*², indicating its superior predictive performance over the other models. By adopting the prediction model introduced in this study, it can be found that it has excellent performance in prediction accuracy and fitting effect, and can be used for global optimization algorithms for optimization.

**Fig 10:**
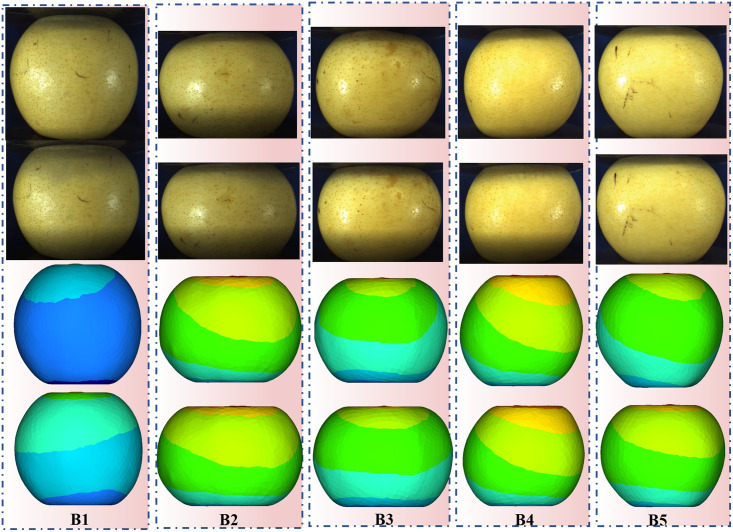
The main deformation modes of pears used for testing.

As shown in Fig 10, the pear first undergoes compression in the contact surface area with the moving pressure plate. As the compression displacement progresses, the end of the pear undergoes significant material shrinkage, and there is a slight expansion in the transverse direction of the pear. During the experiment, there will be a small liquid overflow. The experimental results are compared with the finite element simulation results calibrated by material mechanics parameters, and the two are consistent in the main deformation mode and triggered deformation sequence. The simulation model obtained through material mechanics parameter identification can simulate the main characteristics of the outer contour compression deformation of Huangguan pear.

**Fig 11:**
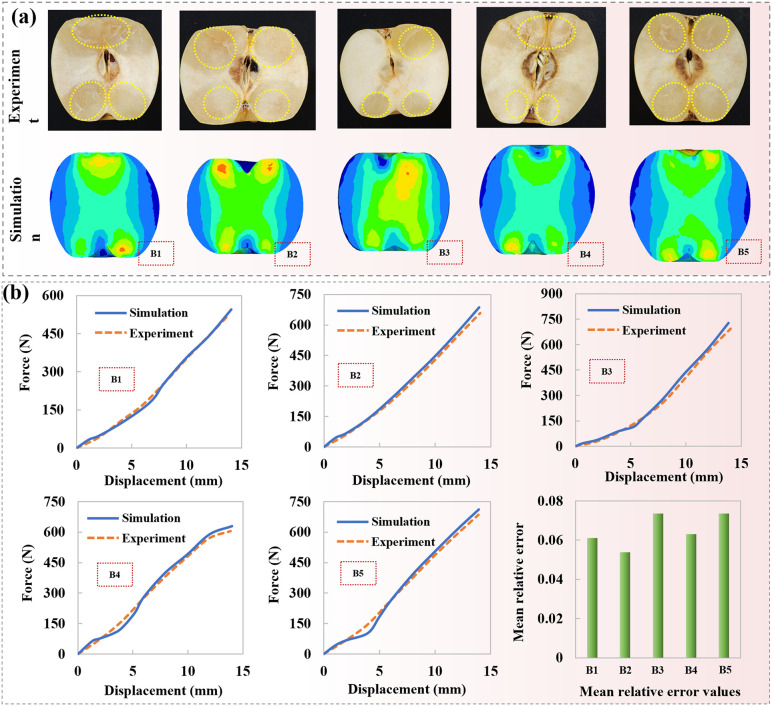
Parameter identification results and verification: (a) Compression deformation profiles of different pears;(b) Compression load and accuracy analysis.

As shown in Fig 11, the internal deformation modes of the validation samples in compression experiments and the simulation model are highly consistent. To further describe the compression deformations within the fruit, the pear is longitudinally cut open to see that both in the experiment and simulation there are uniform crushing deformations zones. Discoloration of pear flesh following bruising mainly occurs due to the enzymatic browning reactions caused by cell damage. Mechanical injury causes phenolic substrates that were initially compartmentalized in various areas of the cells to interact with polyphenol oxidase [50]. The fact that the experimental and simulated deformation zones are nearly identical suggests that the calibrated model is a reliable tool for predicting where and how much bruising occurs, allowing the estimation of quality loss during the postharvest stage in commercial supply chains. In practical storage and processing control, the effective reduction of browning can be achieved by adding an antioxidant or by altering pH to inhibit the activity of PPO. Besides, the analysis of compression load-displacement curves of the experiment and simulation shows that the trends of the curves are approximately similar.

The mean error is used to quantify the difference between the two. The average error remains below 0.08, confirming that the proposed identification method can reliably determine the constitutive parameters of pear tissue. The identified viscoelastic parameters satisfy the physical constraints expected for biological materials, with the instantaneous shear modulus consistently larger than the long-term shear modulus, and the bulk modulus greater than both. This consistency across multiple samples indicates that the identification process not only fits the force displacement curves but also respects the underlying material behavior, enhancing the credibility of the identified parameters for engineering applications. The optimized system framework is utilized to calibrate the fruit damage model, achieving high accuracy through minimal physical testing. It is effective to simulate the small deformation damage of pears by using a viscoelastic constitutive model. Of course, the effectiveness of using viscoelastic constitutive models to simulate the mechanical behavior of fruits has also been mentioned in existing literature [51–54]. Beyond pear tissue, this technique can be extended to mechanical parameter identification for other fruit varieties. The approach is efficient and effective in constitutive model parameter acquisition, and it reduces experimental costs. From a food processing engineering perspective, the ability to obtain reliable viscoelastic parameters from limited physical tests is particularly valuable. These parameters can be incorporated into simulation models used to evaluate equipment designs before physical prototypes are built, reducing development time and improving processing outcomes. The current framework assumes homogeneous and isotropic material properties for each individual pear, without explicitly accounting for biological variability across fruits. In practice, factors such as maturity stage, harvest time, and storage conditions can significantly affect the mechanical behavior of pear tissue. Future work will extend the proposed methodology by incorporating probabilistic modeling techniques, such as Bayesian inversion or variational inference, to quantify parameter uncertainty and enhance the robustness of the identification framework across a broader range of fruit conditions. Such developments would further improve the practical applicability of the approach in real-world food processing environments.

## 4. Conclusions

The present work proposes an optimization-based framework to calibrate a minor-damage model of Huangguan pears subjected to mechanical loading. Combining experimental measurement, finite element simulations, and inverse parameter identification, it offers an effective way of determining the mechanical properties of fruits without the constraints associated with the conventional destructive testing methods. In the CNN-BiLSTM-Attention prediction model tasks, the model achieves *MAPE* values between 0.05 and 0.08 and *R*² values between 0.90 and 0.96, indicating satisfactory predictive ability. A new global optimization algorithm was developed based on an improved genetic algorithm (IGA). IGA also shows significantly better results on the benchmark functions compared to standard GA and PSO, with the best reduction in fitness value reaching 99.95%. This increased optimization ability allows identifying important viscoelastic constitutive parameters, such as the instantaneous shear modulus, long-term shear modulus, relaxation rate constant, and bulk modulus, with confidence. Experimental validation through independent experiments proved that the predicted deformation modes and load-displacement responses based on identified parameters have good agreement with experiment, with an error of less than 0.08. These results confirm the validity, practicality, and reliability of the developed methodology. The suggested strategy is both computationally efficient and experimentally economical when characterizing the mechanical behavior of fruit tissues. The framework has less reliance on large-scale physical testing and has significant potential to be extended to other areas of use, including the prediction of slight damage to fruits, constitutive modelling, and the design and optimisation of equipment used for harvesting, handling, and transporting fruits.

## Supporting information

S1 Video Experimental video (A1-A5). (ZIP)

S2 Video Experimental video (a1 ~ a5). (ZIP)
